# Test Your Knowledge: Ten Questions about Influenza: This quiz is related to a Policy Forum (DOI:10.1371/journal.pmed.0020359) in the December 2005 issue

**DOI:** 10.1371/journal.pmed.0020425

**Published:** 2005-12-27

**Authors:** Gavin Yamey

Question 1. In the United Kingdom, roughly how many patients with influenza-like illness are hospitalized each year?
  0.1%
  1%
  10%
  In the UK, 1.3% of patients with influenza-like illness are hospitalized each year (95% confidence interval, 0.6%–2.6%) [1].
HansenL2004InfluenzaClin Evid20041095110215865706
Question 2. In tropical areas, when does influenza activity typically peak?
  Between late December and early March
  Between May and September
  There is no temporal peak during the year
  In tropical areas, there is no temporal peak in influenza activity [1]. In contrast, in temperate areas, influenza typically peaks between May and September in the southern hemisphere and between late December and early March in the northern hemisphere.
BridgesCBFukudaKUyekiTMCoxNJBridgesCBet al2002Prevention and control of influenza: Recommendations of the Advisory Committee on Immunization Practices (ACIP)MMWR Recomm Rep51(RR-3)13112002171
Question 3. For how long is an otherwise healthy adult with symptomatic influenza infectious to others?
  Only while symptoms are present
  For about one day before the onset of the symptoms until about five days thereafter
  For about three days before the onset of the symptoms until about seven days thereafter
  People with influenza are infectious to others as long as they are shedding virus. Adults with influenza who are otherwise healthy shed virus from about one day before the onset of symptoms until about five days thereafter [1]. It is unclear if people with asymptomatic influenza infection transmit the virus.
FlemingD2005Influenza pandemics and avian fluBMJ331106610691626949410.1136/bmj.331.7524.1066PMC1283192
Question 4. Which of the following is true about avian flu (flu caused by avian viruses) at the time that this quiz was written (December 5, 2005)?
  There is currently no good evidence of human-to-human spread, but flu experts are concerned that such spread could occur in the future
  It is always fatal
  Symptoms are confined to the respiratory tract
  It has only occurred in Asia
  To date, there has been no documented case in which avian flu has spread from human to human [1]. Avian flu is not always fatal; for example, in the first described outbreak of avian H5N1 infection in Hong Kong, six out of the 18 affected people died [1]. In 2003, the avian H7N7 virus was detected in the Netherlands in 86 humans who handled affected poultry and in three of their family members [2]. Of these 89 patients, 78 presented with conjunctivitis, five with conjunctivitis and influenza-like illness, two with influenza-like illness, and four did not fit the case definitions. There was one fatality, which was due to pneumonia in combination with acute respiratory distress syndrome.
FlemingD2005Influenza pandemics and avian fluBMJ331106610691626949410.1136/bmj.331.7524.1066PMC1283192 FouchierRASchneebergerPMRozendaalFWBroekmanJMKeminkSAet al2004Avian influenza: A virus (H7N7) associated with human conjunctivitis and a fatal case of acute respiratory distress syndromeProc Natl Acad Sci U S A101135613611474502010.1073/pnas.0308352100PMC337057
Question 5. Which of the following groups does the United States Centers for Disease Control and Prevention recommend should receive the inactivated influenza vaccine every year?
  Children under six months of age
  Everyone over 40 years of age
  Residents of long-term care facilities
  People who live in long-term care facilities are at high risk for flu complications, so the US Centers for Disease Control and Prevention (CDC) recommends that they get vaccinated each year (http://www.cdc.gov/flu/protect/keyfacts.htm). People aged 65 years and older are at high risk for flu complications, so the CDC states that they should be vaccinated (http://www.cdc.gov/flu/protect/keyfacts.htm). In addition, since almost one-third of people 50–64 years of age in the US have one or more medical conditions that place them at increased risk for serious flu complications, vaccination is recommended for all persons aged 50–64 years (http://www.cdc.gov/flu/protect/keyfacts.htm). The vaccine is not approved for use in children younger than six months of age.
Question 6. Which of the following is an absolute contraindication to receiving inactivated influenza vaccination?
  Being HIV positive
  Pregnancy
  Hypersensitivity to eggs
  One of the indications for receiving influenza vaccination is an underlying chronic medical illness, including HIV/AIDS, and the CDC states that “people with HIV/AIDS are considered at increased risk from serious influenza-related complications and should be vaccinated” (http://www.cdc.gov/flu/protect/hiv-flu.htm). The CDC states that people with HIV/AIDS should not receive the live attenuated flu vaccine (sold commercially as FluMist).
  Pregnancy is not an absolute contraindication to receiving influenza vaccination, but hypersensitivity to eggs is a contraindication because flu vaccine is produced from viral material that is grown on hen eggs [1].
FlemingD2005Influenza pandemics and avian fluBMJ331106610691626949410.1136/bmj.331.7524.1066PMC1283192
Question 7. Which of the following best reflects the clinical evidence on influenza vaccination?
  There is no evidence that vaccination reduces the incidence of pneumonia
  Observational studies suggest that vaccination reduces the incidence of pneumonia
  There is very good evidence from randomized controlled trials (RCTs) that vaccination reduces the incidence of pneumonia
  In a systematic review of studies on influenza vaccination, Loeb found no RCTs that assessed the effects of influenza vaccination on preventing community-acquired pneumonia [1]. However, he found one systematic review of 20 cohort studies that compared influenza vaccination against no vaccination [2]. The review found that vaccination significantly reduced the incidence of pneumonia.
LoebM2005Community acquired pneumoniaClin Evid20051862187516135314 GrossPAHermogenesAWSacksHSLauJLevandowskiRA1995The efficacy of influenza vaccine in elderly persons: A meta-analysis and review of the literatureAnn Intern Med123518527766149710.7326/0003-4819-123-7-199510010-00008
Question 8. Which of the following statements best reflects the evidence on treating influenza with oral oseltamavir (Tamiflu)?
  In high-risk individuals (those aged 65 years and older or those with chronic medical conditions), the drug reduces symptoms by about one day
  There is good evidence that the drug reduces the rate of complications of influenza (including pneumonia)
  In clinical trials, there was no difference in the rates of nausea and vomiting between the drug and the placebo
  In otherwise healthy people with influenza, the drug reduces the duration of symptoms by up to one day
  A systematic review of three RCTs in 937 otherwise healthy people found that the drug reduced the median time to alleviation of symptoms compared with placebo (the difference was −20.69 hours, 95% confidence interval, −33.97– −7.41 hours) [1]. The same review identified five RCTs involving a total of 1,134 high-risk individuals, and it found no significant difference between the drug and the placebo in time for symptom alleviation in this patient group. The review identified one RCT (which did not report on the number of patients) that examined complication rates in otherwise healthy adults treated with drug or placebo; it found no significant difference in the need for antibiotics for complications [1,2]. In clinical trials, nausea and vomiting were significantly more common with the drug than with placebo [3,4].
CooperNJSuttonAJAbramsKRWailooATurnerDet al2003Effectiveness of neuraminidase inhibitors in treatment and prevention of influenza A and B: Systematic review and meta-analyses of randomised controlled trialsBMJ32612351279173510.1136/bmj.326.7401.1235PMC161558 HansenL2004InfluenzaClin Evid2004109510215865706 TreanorJJHaydenFGVroomanPSBarbarashRBettisRet al2000Efficacy and safety of the oral neuraminidase inhibitor oseltamivir in treating acute influenza: A randomized controlled trialJAMA283101610241069706110.1001/jama.283.8.1016 NicholsonKGAokiFYOsterhausADTrottierSCarewiczOet al2000Efficacy and safety of oseltamivir in treatment of acute influenza: A randomized controlled trialLancet355184518501086643910.1016/s0140-6736(00)02288-1
Question 9. Which of the following statements best reflects the evidence on treating influenza with oral amantadine or rimantidine?
  These drugs have specific antiviral activity against influenza A viruses, but not B viruses
  In people with influenza A, these drugs reduce the duration of symptoms by about three days
  There is good evidence from several RCTs that these drugs prevent serious complications of influenza, such as pneumonia
  In vitro, these two drugs have specific antiviral activity against influenza A viruses, but not B viruses [1]. One systematic review [2] and three additional RCTs [3–5] found that the drugs reduce the duration of symptoms by about one day compared with placebo in people with influenza A. One systematic review found no evidence from RCTs that either of these drugs can prevent serious complications of influenza [6].
TominackRLHaydenFG1987Rimantadine hydrochloride and amantadine hydrochloride use in influenza A virus infectionsInfect Dis Clin North Am14594783332798 JeffersonTODemicheliVDeeksJJRivettiD2002Amantadine and rimantadine for preventing and treating influenza A in adultsCochrane Database Syst Rev2002CD00116910.1002/14651858.CD00116912137620 BakerLMShockMPIezzoniDG1969The therapeutic efficacy of Symmetrel (amantadine hydrochloride) in naturally occurring influenza A2 respiratory illnessJ Am Osteopath Assoc68124412504899605 GalbraithAWSchildGCPotterCWWatsonGI1973The therapeutic effect of amantadine in influenza occurring during the winter of 1971–1972 assessed by double-blind studyJ R Coll Gen Pract2334374574716PMC2156817 WaltersHEPaulshockM1970Therapeutic efficacy of amantadine HClMo Med671761795415106 HansenL2004InfluenzaClin Evid2004109510215865706
Question 10. Which of the following statements best reflects the evidence on treating influenza with inhaled zanamavir (Relenza)?
  The treatment is no better than placebo at reducing duration of symptoms
  There is no clinical evidence to suggest any major side effects from the drug
  The drug has activity against influenza A and B viruses
  In vitro studies have shown that zanamavir has antiviral activity against both influenza A and influenza B viruses [1]. There is observational evidence that the inhaled drug may be associated with bronchospasm and worsening of underlying respiratory disease [2,3]. A systematic review of the evidence found that the drug reduces the duration of symptoms by about one day compared with placebo in people with influenza A or B [3].
WoodsJMBethellRCCoatesJAHealyNHiscoxSAet al19934-guanidino-2,4-dideoxy-2,3-dehydro-N-acetylneuraminic acid is a highly effective inhibitor of both the sialidase (neuraminidase) and growth of a wide range of influenza A and B viruses in vitroAntimicrob Agents Chemother3714731479836337910.1128/aac.37.7.1473PMC187997 HenneyJE2000Revised labeling for zanamivirJAMA284123410.1001/jama.284.10.1234-jfd00007-3-110979089 HansenL2004InfluenzaClin Evid20041095110215865706
